# Miscellaneous

**Published:** 1886-03

**Authors:** 


### MISCELLANEOUS ITEMS.

**Priests** were the first physicians of Rome.

**Asdepiades** practiced medicine in Rome ninety years before the Christian era.

**Praxagorias**, the successor of Hippocrates, first made observation of the pulse.

**Melampus** was the first Greek physician. Hippocrates flourished 480 B. C.

**G. R. F.**—You had better send a draft; that will cost you less and be perfectly safe.

**One** hundred years before the Christian era the Greeks did not understand how to tie an artery.

**Hesophilus** was the first to reduce dislocation of the shoulder, and the first founder of scientific anatomy.

**Cyrus and Darius** sent to Egypt for physicians, and there the positive treatment was practiced.

**Physic** is said to beat "faith cure," according to *Texas Siftings*, because it always has the inside track.

**The** best graham flour that we know of is manufactured by Mr. Ferdinand Schumacher, of Akron, Ohio.

**Don't** send us any FIVE and TEN cent stamps. Only *one* and *two* cents are good for payment to the JOURNAL.

**The** *Southern California Practitioner* records a case of the birth of a child, at four months, which resembled a dog, the mother having been frightened by the animal some time before.

**Dio Lewis** says an Englishman, without observing the laws of exercise or sleep, will digest an enormous dinner and preserve his stomach, because of the two hours chat and good fellowship afterwards.

**A Los Angeles** darkey came running into our office a few days since and said: "Doctor, come and see Annie Mariar quick, she is having an awful attack of the harmonia." Questioning developed the fact that he thought his child had Pneumonia.

**Scriptural**.—A man in the West is selling off, and advertises his stock at "ruinous discount; no such sacrifices have been made since the days of Abraham and Isaac." Another advertiser, a fruit-dealer in Boston, explains that his apples "are selling like manna in the desert; nothing has been known like it since the Israelites crossed the Red Sea!"

**Hippocrates** taught that observation was the foundation of reasoning, and he held that the foundation of all disease was in the fluids; but he had no idea of the circulation of the fluids in the human body.

**Dr. Shrady**, the editor of the *Medical Record,* laughs at the recent hydrophobia scare: he declares that there have only been three cases of genuine hydrophobia reported in the United States in the last ten years, and that he does not believe there has ever been a genuine case in the State of New Jersey.

**We** NOTICE that Dr. Austin Flint, Sr., is tó deliver the address on medicines before the British Medical Association in England this year. We suppose he will, of course, take a copy of the Buffalo Medical Journal, for March 1851, as a proof of his extraordinary erudition as a medical expert. So much has transpired since then to make him famous!!!

**The** State Board of Charities of Illinois have investigated the Asylum at Chicago under the city charge, and found them as badly fed and treated as they have just discovered them to be in Newark. As long as mere politicians have the care of these institutions there is no protection for the insane. It is an absolute abuse to allow a party to have anything to do with the insane.

**The** Hindoos never made the medical profession famous; but we do not learn that the nation suffered for want of physicians. Nor do we find the Chinese suffering for want of doctoring the body. Neither did the Jews ever produce a school of medicine, but they have always been a healthy people, for all that. They have never eaten "hog."

**The** question is being mooted abroad whether the feminine brain is equal to the strain of the medical profession. In England, according to the census of 1881, the number of women physicians was twenty-five. From 1880 to 1884 eight were placed in the lunatic asylum. These facts are important, if reliable, but it is far from the truth in this country and probably is so in Great Britain.

**A Physician** in New York (Dr. L. G. Doane) says that people become insane from doing one thing continually. Thus an actor acting one part many months is liable to become a maniac. A telegraph operator sending stock reports is liable to become maniacal. The continual use of the brain or one function of the brain leads in many instances to insanity, "Variety is the spice of life." Verb Sap. Monotony, Dr. Doane says, leads to melancholia, a mild form of insanity, now recognized and treated.

